# Efficiency evaluation of commercial banks in Pakistan: A slacks-based measure Super-SBM approach with bad output (Non-performing loans)

**DOI:** 10.1371/journal.pone.0270406

**Published:** 2022-07-12

**Authors:** Wasi Ul Hassan Shah, Gang Hao, Hong Yan, Rizwana Yasmeen

**Affiliations:** 1 School of Management, Zhejiang Shuren University, Hangzhou, China; 2 Department of Management Sciences, City University of Hong Kong, Hong Kong, China; 3 School of Economics and Management, Panzhihua University, Panzhihua, Sichuan China; Foshan University, CHINA

## Abstract

According to recent figures from the State Bank of Pakistan (SBP), since 2006, commercial banks’ non-performing loans (NPLs) have significantly risen. To this end, the primary objective of this research is to explore the impact of NPLs on the operational efficiency of commercial banks in Pakistan. NPLs were incorporated as bad output in the efficiency estimation of 24 CBs for the period 2006–2017. This study employs the data envelopment analysis (DEA) Super-SBM with the undesirable output for the efficiency evaluation of CBs. To test the robustness of our results, we used two different input-output bundles (model A and model B). The findings show a significant difference exists between the results estimated with and without undesirable output. Furthermore, the results of super-efficiency estimation rank the most efficient CB for the study period and distinguish it from other efficient DMUs. Models A and B show that foreign banks are always more efficient than domestic banks, while private CBs have higher efficiency scores than public CBs in domestic banking. In addition, the big five CBs show mixed findings, as in model A, they were more efficient than other domestic CBs, while in model B were less efficient. In the second stage of the empirical study, we use the system GMM to examine the impact of NPLs, bank size, and net interest margin on CBs efficiency. We discovered that NPLs have a negative and significant effect on banking efficiency, whereas bank size and net interest margin positively affect the efficiency of commercial banks in Pakistan.

## 1. Introduction

Pakistan’s commercial banking industry witnessed a consistent increase in its Assets and profitability from the last two decades, as total assets of the banking sector grew from Rs 22,120.46 billion in 2019 to Rs 25,069.06 billion in 2020, showing an increase of 13.33 percent [1]. Amin (2007) [2] argues that government liberalization and privatization policy does not improve the financial health of CBs in Pakistan due to over-employment and debt burden. Commercial banks play a critical role in the financial sector by supplying funds to achieve economic development goals [3]. Schumpeter [4] recognized that commercial banks are essential sources of long-term financial investments. CBs are the backbone of the banking system because they are a major source of lending capital to the private sector [5]. The developed financial sector has massive potential to improve and sustain the economic growth of any country or region [6]. Indeed, these commercial banks contribute widely to financial capital mobilization in any economy. To maintain the banking system’s stability, operational efficiency is essential in keeping the CBs profitable and healthy, as Belke et al. (2016) [7] argue that more efficient CBs foster regional economic growth.

The performance of CBs is critical in proposing policy-making for decision-makers. There are numerous techniques used to measure the performance of CBs; two renowned methods for efficiency evaluation are parametric (stochastic frontier analysis) and non-parametric (data envelopment analysis). DEA is an extensively used technique in evaluating banks around the Globe [8]. Tone (2004) [9] introduced an (SBM) model in DEA, which could incorporate bad output to gauge more accurate efficiency results. Each particular industry has its undesirable outcomes in the production process; usually, CBs consider NPLs as bad output, affecting the efficiency of any specific DMU.

The study of Negera (2012) [10] explains that a low ratio of Non-performing loans (NPLs) provides solidity in any economy’s financial system, as NPLs can reduce the lending capacity of CBs, which causes a lack of financial support to many potential projects. Further, it influences bank profitability as loans are the primary source of revenue for any CB, and large NPLs can reduce the liquidity of bank funds. NPLs can affect the whole market economy [11, 12]; therefore, they are essential in commercial bank efficiency. Partovi & Matousek [13] demonstrated that, on average, CBs with a higher rate of NPLs are less efficient in the Turkish banking industry. Numerous research studies described the importance of NPLs in banks’ performance and proved that NPLs negatively impact the efficiency of CBs because they deteriorate asset quality [14, 15]. Few Studies used NPLs as a control variable in their performance analysis [16–18]. In comparison, most researchers used NPLs as bad output in their DEA models [19–23].

**Fig 1:**
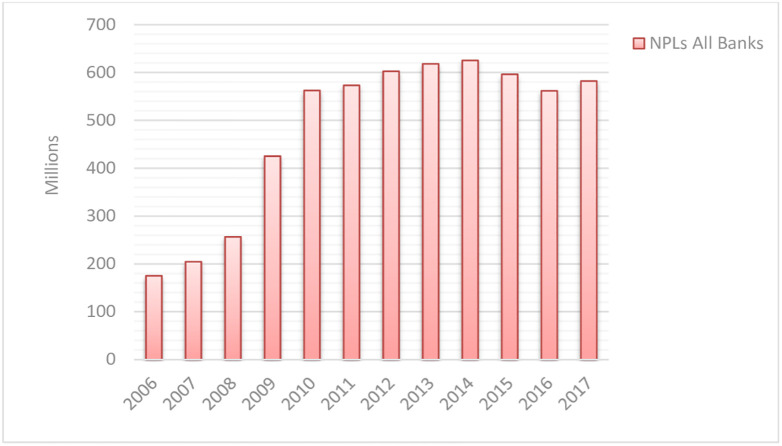
Growth of NPLs in Pakistan CBs industry for 12 years (2006–2017).

In the recent decade (2006–2017), NPLs of Pakistan’s commercial banking industry increased rapidly, as they grew to .582 billion in 2017 from .175 billion in 2006 (see Fig 1).

**Fig 2:**
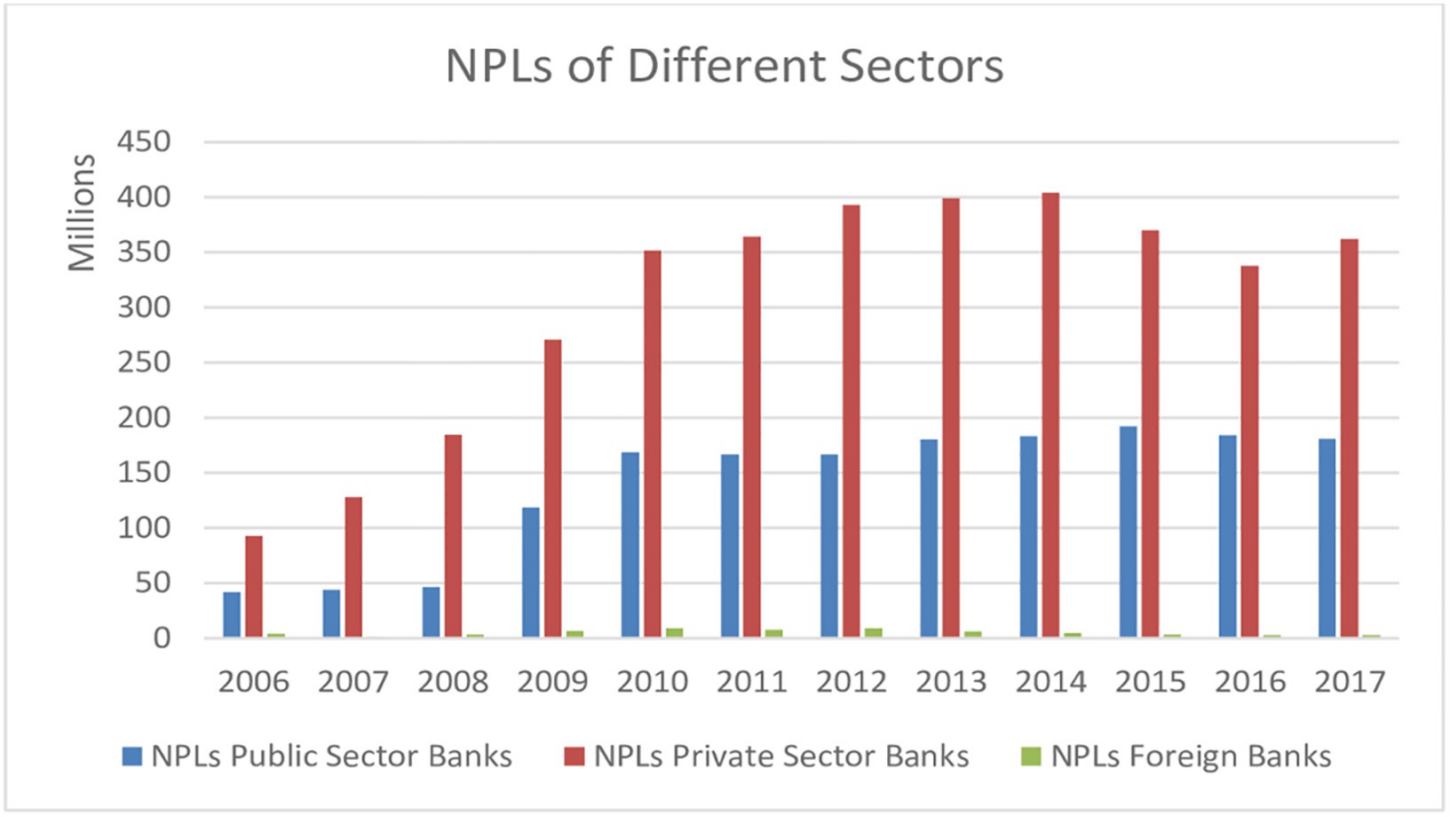
Growth of NPLs in different sectors of Pakistan’s CBs industry for the period (2006–2017).

Further elaborating the different commercial banking sectors, it was witnessed that 97.5% of total NPLs were borrowed from local banks in the year 2006, which increased to 99.5% in 2017, while the percentage of NPLs taken from foreign banks was only 0.5% in 2017. Local CBs comprise public and private CBs; statistics indicate that at the end of 2017 share of private CBs in NPLs of Local banks was 62.5%; Public CBs count for 31.2% while reaming 6.3% of NPLs were taken from specialized banks (see Fig 2). This phenomenon indicates the constant pressure of growing NPLs on Pakistan’s commercial banking, which could affect the operational efficiency of CBs. Literature advocates that although many studies (see Table 1) evaluate the efficiency and productivity of Pakistan’s commercial banking industry over different periods. None of the researchers incorporates the NPLs as bad output in their DEA input-output bundles and evaluates the impact of NPLs on the efficiency of CBs. Karou [24] explains that without considering undesirable output in the performance evaluation process could affect the efficiency scores of DMUs. Therefore, enormous growth in the NPLs of the Pakistan banking industry has great concern not only for central bank authorities but also for investors and stockholders of private CBs. Further bank size and net interest margin are also critical contextual variables that impact the banking efficiency of the financial sector in any economy.

**Table 1:** Studies employed DEA to measure efficiencies of commercial banks in Pakistan.

Authors	Title of Study	Use NPLs
Ali Rizvi [77]	Post-liberalization efficiency and productivity of the banking sector in Pakistan	NO
Iimi [78]	Efficiency in the Pakistani banking industry: Empirical evidence after the structural reform in the late 1990s	NO
Akhtar [79]	X-efficiency Analysis of Commercial Banks in Pakistan: A Preliminary Investigation	NO
Ataullah et al., [80]	Financial liberalization and bank efficiency: A comparative analysis of India and Pakistan	NO
Kiani [81]	A Comparison of Domestic Vs. Foreign Banks Using Stochastic Frontier Approach	NO
Qayyum et al., [82]	X-efficiency, scale economies, technological progress and competition: A case of the banking sector in Pakistan	NO
Akmal et al., [83]	Technical efficiency of the banking sector in Pakistan	NO
Ahmed et al., [84]	Efficiency dynamics and financial reforms: A case study of Pakistani banks	NO
Akhtar [85]	X-efficiency analysis of commercial banks in Pakistan: A preliminary investigation	NO
Ur Rehman et al., [86]	Efficiencies of the Pakistani banking sector: A comparative study	NO
Nazir et al., [87]	The Impact of Financial Restructuring on the Performance of Pakistani Banks: A DEA Approach	NO
Ahmad [88]	Financial reforms and banking efficiency: Case of Pakistan	NO
Haque et al., [89]	The efficiency of Banks in Pakistan: A Non-Parametric Approach	NO
Qayyum et al., [90]	Technical Efficiency of Pakistani Banks: An Application of Bootstrapped Data Envelopment Analysis Approach	NO
Mustafa et al., [91]	Efficiency Change in Pakistan Commercial Banking Sector: A Pre and Post Digital-Restructuring Analysis	NO
Zhu and Wasi [92]	A Cross-Country Comparison of Operational Efficiency between Chinese and Pakistani Commercial Banking Industries	NO
Zhu and Wasi [93]	Efficiency and productivity analysis of Pakistan banking industry: A DEA approach	NO

To this end, our research contributes to the literature in several ways; firstly, it incorporates the NPLs in the efficiency estimation of CBs to gauge the impact of bad-output. Secondly, the super-efficiency model (SBM) will distinguish and rank the most efficient DMU in Pakistan’s commercial banking for 12 years (2006–2017). Thirdly this research explains whether changing the input-output bundles affect the super efficiency scores of evaluated DMUs. Fourthly, this research differentiates commercial banking sectors (public, private and foreign CBs), elaborates the most efficient sector, and further separates the efficiency of big 5 CBs from other domestic CBs. Finally, system GMM reveals the association of NPLs, bank size, and net interest margin with CBs efficiency. The rest of the paper is organized as section 2 explains the study’s brief, relevant literature; Section 3 describes the methodological part, data, and models selection. While results discussion and conclusion are presented in sections 4 and 5, respectively.

## 2. Literature review

The study by Charnes [25] introduced a non-parametric technique to measure the relative efficiency of different decision-making units (DMUs), known as data envelopment analysis (DEA). Their conventional model (CCR) with the constant return to scale assumption was further modified by Banker [26] with the variable return to scale known as (BCC). Numerous researchers used DEA for the performance evaluation of CBs in different parts of the world.

### 2.1 Banking efficiency

Sherman & Gold [27] were the first to use DEA in the US banking industry to measure the relative efficiency scores of different bank branches. Later on, researchers frequently used conventional (CCR, BCC) models to measure the efficiency in various banking industries around the Globe. Different input-output bundles were used to measure the technical efficiency of several CBs in the USA during (1983–1990) [28–34]. Miller & Noulas [35] applied DEA to evaluate the technical efficiency of 201 commercial banks in the USA between 1984 and 1990 and found that larger and profitable banks are more efficient. However, larger banks are more likely to operate under decreasing return to scale. Berger & Humphrey [15] provide a review of 130 financial institutions, including commercial banks. Following the model proposed by Anthanassopoulos [36], Grifell et al. [37], and Soteriou et al. [38] also studied the productivity and cost-efficiency of CBs. DEA was applied to measure the efficiency of 55 US CBs and revealed that big banks show better performance in terms of profitability while small banks show a better position concerning marketability [39]. A study by Drake [40] measured the efficiency and productivity growth of UK commercial banks and proved that large banks are more X-efficient than their small counterparts. Neal [41] evaluates the efficiency of the Australian banking industry for a specific period and argues that CBs exhibit higher allocative efficiency than technical efficiency. An intermediate approach of input-output selection to measure the efficacies of CBs in developing economies was adopted by [42–46]. Chang et al. [47] measure the efficiency of 20 Chinese state-owned commercial banks and found that their efficiency level is 81 percent; further, service quality positively impacts efficiency and customer satisfaction. Novickytė & Droždz [48] evaluated Lithuanian CBs using the DEA input-oriented model with VRS and CSR assumptions and found that CBs owned by Nordic groups are more efficient than local CBs. Different studies were also conducted for efficiencies estimation of CBs in growing economies of the European Union [49–56].

### 2.2 Non-performing loans and banking efficiency

In the production economies, it is usually assumed that a production unit producing good output will uncontrollably have some bad output [57]. Different DEA models had been proposed to handle the desirable and undesirable output in any production process of a particular industry or sector [58–60]. However, these models have some shortcomings as they transform the bad-output into good and perform the efficiency analysis or input-output oriented. Further radial models ignore the slacks while dealing with undesirable. Therefore, Tone [24] modified its non-oriented, non-radial SBM model Tone [61], extended it with undesirable output, and proposed the SBM-NS model, covering all efficiency measures. Bad outputs yields during the production process vary from industry to industry; usually, the renowned bad output is CO2 omission in industrial production. However, bad outputs in service-oriented sectors are different. Bad service quality and customer complaints are also considered bad output and controllable through better training, operational strategies, and improved performance [62]. Banks produced two types of loans, good and bad (non-performing). Commercial loans are considered non-performing if the borrower is 90 days past the due date. In the commercial banking industry, NPLs are regarded as undesirable output, which influences the efficiency and profitability of CBs. Numerous studies advocate that NPLs are negatively correlated with the stability and efficiency of CBs in different regions of the Globe [14, 15]. Further in the CBs performance evaluation, omitting NPLs from the input-output bundle of the DEA model provides biased efficiency scores [63, 64]. Park & Weber [65] argue that NPLs should be included as a bad-output in the production process to get more authentic and accurate efficiency results.

Considering the importance of NPLs in efficiency evaluations, many researchers incorporate NPLs in their input-output bundles and measure the efficiency of CBs in different countries of the world. Partovi & Matousek [13] used NPLs as undesirable output in their DEA analysis on 44 CBs of Turkey for the year (2002–2014) and explain that NPLs negatively impact bank efficiency and further differentiate the efficiency level ownership structure of the Turkish banking industry. Wang et al. [66] took NPLs as a bad output in their DEA network Model applied on 16 Chinese CBs (2003–2011) and revealed that the overall efficiency of Chinese CBs has improved after the reforms State-owned CBs are most efficient among all. Fukuyama & Weber [22] develop a dynamic network DEA model to measure the efficiency of Japanese CBs and incorporate NPLs as bad output in their proposed model to estimate the inefficiencies of CBs for a specific period. Hajialiakbari et al. [21] measured the effect of NPLs on the technical efficiency of Iranian government banks. They concluded that NPLs have a non-linear negative impact on the TE of state-owned CBs. Barros [67] evaluated the efficiency of Japanese CBs for the years (2002–2007) and included undesirable output in the estimation process, and revealed that NPLs (bad-output) are the burden on the performance of CBs in Japan. Many other studies also included NPLs as undesirable output in their efficiency evaluation of CBs [68, 69].

Conventional DEA models do not rank the efficient DMUs, while efficiency results often show more than one efficient DMUs. Andersen [70] proposed a DEA model to rank the efficient units (DMUs). Later, Tone [61] introduced a slack-based measure (SBM) and further modified slack base measure for super-efficiency [71]. Tone [24] presented a revised model for more precise efficiency estimation incorporating the undesirable outputs in the Super-SBM. Noura et al. [72] proposed a more effective DEA super-efficiency model and divided the inputs and outputs into desirable and undesirable. Zimková [73] estimates the technical and super efficiency of CBs in Slovakia using BCC, SBM, and Super SBM. Literature advocates that numerous research studies used Super-SBM to measure the efficiency of CBs in different parts of the world [74–76].

### 2.3 Banking efficiency in Pakistan

Pakistan’s commercial banking industry mainly consists of local and foreign banks. Local banks are further divided into public, private, and specialized banks. Banking statistics show that return on equity (ROE) increased from 13.27 percent in fiscal year 2019 to 16.99 percent in the fiscal year 2020, while return on assets (ROA) increased from 0.83 percent in the fiscal year 2019 to 1.07 percent in the fiscal year 2020. Many researchers have estimated efficiencies (cost, operational, technical, profit, and revenue) of CBs in Pakistan. SBP [1] explains the continued growth of NPLs in the commercial banking industry of Pakistan for the 12 years (2006–2017) (see Figs 1 & 2). However, the literature of efficiency estimation (see Table 1) indicates that none of the researchers incorporated the NPLs in their DEA models nor applied the super-SBM model to benchmark the efficient DMU in the banking industry. Further, although past studies proved that bank size is positively associated with banking efficiency, no research distinguishes the efficiency of big 5 Pakistani CBs from remaining domestic CBs.

### 2.4 Association of NPLs, bank size and net interest margin with banking efficiency

Kwan et al. [94] argued that none performing loans play an important role in banking inefficiency. Further, through empirical analysis, they proved that a significant negative relationship between the NPLs and cost efficiency of CBs exists. In addition, more researchers demonstrated that banks inefficiency is correlated with NPLs [95–97]. NPLs have a negative and significant impact on banking efficiency; a decrease in NPLs level improves the growth of cost efficiency of CBs in Malaysia and Singapore [98]. Stephen [99] used data of 16 CBs for years (2007–2015) and found that bank profitability is negatively associated with NPLs. Further studies emphasize that banking efficiency is positively associated with the scale of banks [100–102]. Similarly, Kovner et al. [103] concluded that bank sizes in the US positively impact cost efficiency. At the same time, many other studies proved that bank size positively and significantly influences the operational efficiency of banks and financial institutions [104–106]. Past studies advocate that bank size is usually measured through the total Assets of that particular bank. Samsonova [107] argues that net interest margin is the best financial indicator of firm performance and is significantly related to banks’ operational efficiency. Bandaranayake [108] took NIM as an efficiency indicator to determine the factor influencing bank efficiency in Sri Lankan banking; however, not much literature was found that measures the association of super efficiency of commercial banking with NPLs, bank size, and NIM.

## 3. Data and methodology

DEA is a popular efficiency estimation technique because it doesn’t make any assumptions about the shape of the frontier surface or how a DMU’s internal operations work. DEA is extensively used to gauge the operational efficiency of financial institutions, particularly banks.

### 3.1 Super-SBM with undesirable output

Unlike conventional CCR and BCC models, Tone [61] SBM model directly puts slacks into the objective function to counter the input-output slacks problem. SBM is a non-radial non-oriented model; it avoids the radial and oriented deviation and gauges the most authentic efficiency results. Efficiency estimation from the conventional DEA model reveals that usually, there are more than 1 DMUs that score unity; therefore, efficient units cannot be distinguished. To handle this issue, Tone [71] proposed a Super-SBM model, where the efficiency score of efficient DMUs could exceed one and rank them accordingly. Every production unit yields controllable and uncontrollable undesirable outcomes along with good outputs. In modern banking systems, NPLs are considered as bad output. This study uses the Super-SBM model with undesirable output based on research by [109], to check the effect of NPLs on the efficiency evaluation of 24 CBs in Pakistan.

$x\in R^{m},y^{g}\in R^{s_{2}},y^{b}\in R^{s_{2}}$. The matrices *X*, *Y*^*g*^, *Y*^*b*^ are defined as follows

$$\begin{array}{rr} & X=\left[x_{1},x_{2},\cdots ,x_{n}\right]\in R^{m\times n},\\ & Y^{g}=\left[y_{1}^{g},y_{2}^{g},\cdots y_{n}^{g}\right]\in R^{s_{1}\times n},\\ & Y^{b}=\left[y_{1}^{b},y_{2}^{b},\cdots y_{n}^{b}\right]\in R^{s_{2}\times n}, \end{array}$$

Assume that, *X* > 0, *Y*^8^ > 0, *Y*^*b*^ > 0. Then the production possibility set *P* is defined by *P* = {(*x*, *y*^*g*^, *y*^*b*^) | *x* ≥ *xλ*, *y*^*g*^ ≤ *y*^*g*^*λ*, *y*^*b*^ ≥ *y*^*b*^*λ*, *λ* ≥ 0}

A specific DMU $\left(x_{0},y_{0}^{g},y_{0}^{b}\right)$ is expressed as

$$\begin{array}{rr} & x_{0}=X\lambda +S^{-}\\ & y_{0}^{g}=Y^{g}\lambda -S^{g}\\ & y_{0}^{b}=Y^{b}\lambda +S^{b}\\ & S^{-}>0,S^{g}>0,S^{b}>0 \end{array}$$

The vectors $S^{-}\in R^{m},S^{g}\in R^{s_{1}}$ and $S^{b}\in R^{s_{2}}$ Present slacks in inputs, good outputs, and bad outputs, respectively. *M*, *s*_1_ and *s*_2_ show the number of factors for inputs, good outputs and bad outputs. "*S*^−^" Indicates that the actual input resource is more than frontier investment. "*S^g^*" Shows that the good output produced in the actual operation is less than frontier desirable output. "*S*^*b*^" means that the actual undesirable output level is greater than the leading edge of the undesirable output level. λ is the intensity vector. In the presence of bad output, DMU $\left(x_{0},y_{0}^{g},y_{0}^{b}\right)$ is only SBM-efficient if and only if there are no excessive input, no insufficient desirable output, and no surplus undesirable outputs, i.e.


$$S^{-}=0,S^{g}=0,S^{b}=0$$

We discuss the super-efficiency issues under the assumption that the DMU $\left(x_{0},y_{0}^{g},y_{0}^{b}\right)$ is SBM-efficient. The production possibility set *P*′is defined by excluding $\left(x_{0},y_{0}^{g},y_{0}^{b}\right)$ from (*X*, *Y*^*g*^, *Y*^*b*^), as follows:

$$P^{'}=\left\{\left(\overset{‾}{x},{\overset{‾}{y}}^{g},{\overset{‾}{y}}^{b}\right)|\sum_{j=1,j\neq 0}^{n}x_{j}\lambda_{j}\leq \overset{‾}{x},\sum_{j=1,j\neq 0}^{n}y_{j}^{g}\lambda_{j}\geq {\overset{‾}{y}}^{g},\sum_{j=1,j\neq 0}^{n}y_{j}^{b}\lambda_{j}\leq {\overset{‾}{y}}^{b},{\overset{‾}{y}}^{g}\geq 0,\lambda \geq 0\right\}$$

The subset $\overset{‾}{P}$ of *P*′ is defined as

$$\overset{‾}{P}=P^{'}\cap \left\{\overset{‾}{x}\geq x_{0},{\overset{‾}{y}}^{g}\leq y_{0}^{g},{\overset{‾}{y}}^{b}\geq y_{0}^{b}\right\}$$

The following expression shows how Super-SBM deals with undesirable output [61, 109].


$$\delta^{*}=min\frac{\frac{1}{m}\sum_{i=1}^{m}\frac{x_{i}^{-}}{x_{i0}}}{\frac{1}{s_{1}+s_{2}}\left(\sum_{r=1}^{s}\frac{{\overset{‾}{y}}_{r}^{g}}{y_{r0}^{g}}+\sum_{r=1}^{s_{2}}\frac{{\overset{‾}{y}}_{r}^{b}}{y_{r0}^{b}}\right)}$$ (1)

Subject to

$$\begin{array}{rr} & \sum_{j=1,j*0^{n}}^{n}x_{j}\lambda_{j}\leq \overset{‾}{x}\\ & \sum_{j-1,j\neq 0}^{n}y_{j}^{g}\lambda_{j}\geq {\overset{‾}{y}}^{g}\\ & \sum_{j=1,j\neq 0}^{n}y_{j}^{b}\lambda_{j}\leq {\overset{‾}{y}}^{b}\\ & \overset{‾}{x}\geq x_{0},{\overset{‾}{y}}^{g}\leq y_{0}^{g},{\overset{‾}{y}}^{b}\geq y_{0}^{b},{\overset{‾}{y}}^{g}\geq 0,\lambda \geq 0 \end{array}$$ (2)

This study uses Super-SBM DEA Model with undesirable output under constant return to scale (CSR) assumption.

### 3.2 Selection of inputs and outputs

The selection of inputs and outputs variable in DEA is a matter of great concern for researchers. Literature advocates that two approaches are mostly used to measure commercial bank efficiency, namely the production and intermediate approach. Banks are considered service providers in the production approach, where they emphasize operating costs and count deposits as output without considering interest expenses paid on deposit collection. On the contrary, the intermediation approach used deposits as an input variable to produce more bank assets, while all operating costs and interest expenses were used as input. Berger et al. [15] suggest that the production approach is more applicable in branch-level data, while intermediation approaches for bank-level data. Using the intermediation approach, we adopt two different input-output bundles from previous studies of [42, 110] and named them Model A, B in this research. Model A contains two inputs interest expenses, non-interest expenses, and two outputs, net interest income and non-interest income. In contrast, model B includes deposits and the number of employees as inputs, while net loans and non-interest income are output variables. In addition, NPLs were taken as an undesirable output in both Models (see Table 2).

**Table 2:** Model A & B with different set of inputs and outputs.

Models	Inputs	Good Outputs	Bad Outputs
Model A	Interest expenses	Net Interest Income	NPLs
	Non-Interest expenses	Non-Interest Income	
Model B	Total Deposits	Net Loans	NPLs
	Number of Employees	Non-interest Income	

Non-radial and non-oriented Super-SBM model with the assumption of constant returns to scale was employed to estimate the super efficiency scores of 24 CBs, including four public, 18 private, and 2 foreign banks, Max DEA ultra (7.0) was used for data analysis. At the same time, all the data was taken from the financial analysis of Pakistan banking reports (SBP), and the unit was thousand Pakistani Rupees. Descriptive statistics of all the variables are given in Table 3.

**Table 3:** Descriptive statistics of both models.

**Model A**					
Variables	Interest expenses	Non-Interest expenses	Net Interest income	Non-interest Income	Non-performing Loans
Max	68810743	63541423	83067472	32889137	121941324
Min	463007	900071	674512	121978	96511
Average	19455084	15939229	19570430	8217135	22850260
SD	18787753	15895648	20778566	9186235	28452235
**Model B**					
Variables	Total Deposits	Number of Employees	Net Loans	Non-interest income	Non-performing Loans
Max	1998935057	18243	851502420	32889137	121941324
Min	16259737	119	5709278	121978	96511
Average	528590085	6325	262615288	8217135	22850260
SD	523924651	5251	230350523	9186235	28452235

### 3.3 Econometric model

Given the importance of banking efficiency and non-performing loans, the following model is composed as:

$${BE}_{it}={\alpha_{1}BE}_{it-1}+\alpha_{2}{Bank\_Size}_{it}+\alpha_{3}{NIM}_{it}++\alpha_{4}{NPL}_{it}++\alpha_{5}\mu_{it}$$ (3)

Where (*BE*)) indicates the set of banking efficiency of both models. Bank size is denoted by *Bank_Size*, Net interest Margin is signified by *NIM*, and *NPL* is non-performing loans.

In Eq (3), the Generalized Method of Moments (GMM) with two-step is applied for the long-run impact of the concerned variables. The GMM is the best choice with a small-time span (T) and large cross-sections (N) [111]. Moreover, selecting a two-step GMM is more appropriate than the one-step system GMM due to heteroscedasticity and autocorrelation [112, 113]. Furthermore, the beauty of system GMM methods is the independent distribution of the error terms and serially uncorrelated. The lagged values of the variables can use as instruments based on the nature of the variables. The competence of the GMM model can be verified with two diagnostic tests. One is the joint validity of the lagged instrumental variables certified by running the Sargan/Hansen test. Second, the Arellano-Bond test is applied to check for serial correlation in the differenced equations.

## 4. Results and discussion

Section 4.1 presents the super efficiency scores estimated through two different input-output bundles. In contrast, section 4.2 presents an efficiency comparison of Big 5 CBs with other domestic CBs, while section 4.3 presents the econometric results of the model constructed to check the impact of NPLs, bank size, and net interest margin on the banking efficiency of Pakistan.

### 4.1 Efficiencies results of both model A and B

**Fig 3:**
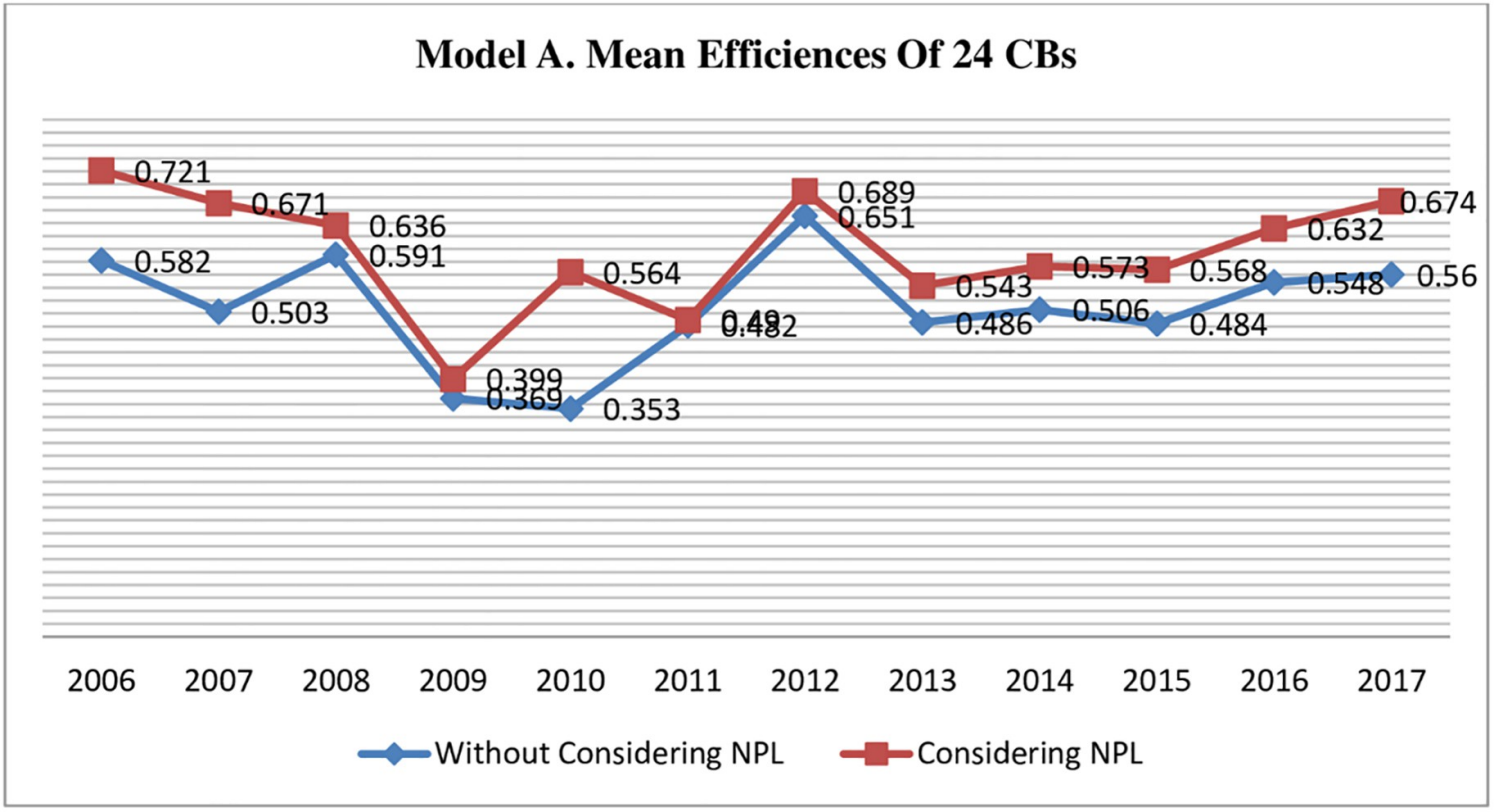
Model, A comparison of mean efficiency scores of all CBs, estimated with and without incorporating undesirable output NPLs.

Table 4 present the results of model A, which indicate the mean efficiency score (considering NPLs) for 12 years (2006–17) is .597 while (without considering NPLs) is 0.510, which suggests that CBs are operating in Pakistan still needs to improve their efficiency level. Moreover, a considerable difference exists among mean efficiency scores calculated considering and without considering NPLs (undesirable output) in the efficiency estimation (see Fig 3). A sector-wise analysis shows us that efficiency scores estimated through incorporating NPLs in Input-Output bundle, foreign CBs perform better than domestic. At the same time, private CBs got better score than public sector CBs (Foreign CBs >Private CBs>Public CBs) = (1.26 >0.554 >0.454). Results indicate that ignoring the NPLs in the efficiency estimation process doesn’t change the mean efficiency level of different sectors of Pakistan CBs; however, mean efficiency scores changed (Foreign CBs >Private CBs>Public CBs) = (1.141 >0.459 > 0.424). Further, it was noticed that DB AG (a foreign CB) was the most efficient CB among all 24 CBs. BAH was ranked one among private, while NBP in public CBs. DB AG (foreign) and NBP (public) don’t change their position when we ignore undesirable output. However, BAH (private) didn’t sustain its position and jumped on to 16th position among all 24 CBs replacing MCB, ranked 1^st^ in private CBs.

**Table 4:** Model A. Efficiency of 24 CBs Considering the undesirable output.

DMU	2006	2007	2008	2009	2010	2011	2012	2013	2014	2015	2016	2017	Mean	Rank
FWB	1.132	1.143	1.164	0.154	0.298	0.392	0.351	0.209	0.169	0.335	0.36	0.192	0.492	13
NBP	0.774	0.613	0.648	0.392	0.53	0.560	0.769	0.456	0.43	0.436	0.492	0.49	0.549	12
BOK	0.397	0.358	0.257	0.207	0.064	0.468	0.607	0.427	0.376	0.415	0.503	0.434	0.376	19
BOP	1.070	1.115	0.189	0.212	0.008	0.009	0.302	0.299	0.309	0.394	0.43	0.503	0.402	17
**Mean public**	**0.843**	**0.807**	**0.564**	**0.241**	**0.223**	**0.355**	**0.507**	**0.347**	**0.321**	**0.395**	**0.446**	**0.404**	**0.454**	
ABL	0.38	0.377	0.414	0.405	0.635	0.552	1.076	0.485	0.602	0.547	0.635	1.013	0.593	10
ASBL	0.455	0.415	0.346	0.242	0.27	0.297	0.433	0.259	0.328	0.333	0.401	0.424	0.350	21
BIP	1.106	0.424	0.84	0.151	0.596	0.239	0.412	0.359	0.61	0.132	0.139	0.209	0.435	15
BAF	0.413	0.376	0.33	0.220	0.352	0.361	0.587	0.388	0.416	0.365	0.486	0.612	0.409	16
BAH	1.031	1.442	1.272	1.164	1.343	1.113	1.139	1.097	1.099	1.062	1.101	1.082	1.162	2
DIBP	1.19	1.419	1.205	0.242	0.346	0.286	0.522	0.348	0.487	0.286	0.448	1.026	0.650	8
FBL	1.004	0.45	1.226	0.26	0.281	0.273	0.478	0.306	0.357	0.335	0.452	0.459	0.490	14
HBL	0.457	0.403	0.500	0.370	0.616	0.608	0.716	0.463	0.551	0.567	0.652	0.747	0.554	11
HMBL	1.275	1.159	0.651	0.414	1.05	0.465	0.653	0.468	0.425	0.492	0.478	0.559	0.674	6
JSBL	0.08	0.324	0.308	0.121	0.193	0.315	1.020	0.573	0.547	0.452	0.491	0.352	0.398	18
MCB	1.204	1.222	1.155	1.233	1.154	1.118	1.124	1.03	1.059	1.08	1.085	0.784	1.104	3
MBL	0.699	0.761	0.351	0.496	1.007	0.466	0.745	0.648	0.6	0.45	0.697	0.948	0.656	7
SBL	0.008	0.099	0.124	0.053	0.238	0.178	0.293	0.181	0.199	0.298	0.406	0.357	0.202	23
SKBL	0.161	0.102	0.079	0.013	0.114	0.157	0.255	0.214	0.296	0.211	0.391	0.397	0.199	24
SRB	0.627	0.354	0.382	0.271	0.307	0.345	0.463	0.337	0.351	0.342	0.347	0.382	0.376	19
SCB	0.644	0.609	1.015	0.387	1.128	0.581	0.877	1.002	1.028	1.063	1.043	1.074	0.871	5
UBL	0.511	0.446	0.528	0.42	0.629	0.625	0.873	0.553	0.638	0.601	0.748	1.007	0.632	9
ABIBP	0.267	0.286	0.214	0.136	0.111	0.224	0.225	0.214	0.255	0.208	0.299	0.256	0.225	22
**Mean private**	**0.639**	**0.592**	**0.608**	**0.366**	**0.576**	**0.456**	**0.66**	**0.496**	**0.547**	**0.49**	**0.572**	**0.649**	**0.554**	
CB NA	1.183	0.909	0.784	0.392	0.794	0.531	1.119	1.259	1.255	1.227	1.249	1.141	0.987	4
DB AG	1.243	1.317	1.291	1.635	1.489	1.625	1.502	1.473	1.386	2.012	1.844	1.732	1.546	1
**Mean foreign**	**1.213**	**1.113**	**1.037**	**1.013**	**1.141**	**1.078**	**1.31**	**1.366**	**1.32**	**1.619**	**1.546**	**1.436**	**1.267**	
**Total Mean**	**0.721**	**0.671**	**0.636**	**0.399**	**0.564**	**0.49**	**0.689**	**0.543**	**0.573**	**0.568**	**0.632**	**0.674**	**0.597**	
**Model A. Efficiency of 24 CBs ignoring undesirable output**														
FWB	0.316	0.509	0.647	0.099	0.073	0.400	0.281	0.206	0.13	0.357	0.336	0.151	0.292	21
NBP	0.923	0.727	0.767	0.437	0.456	0.673	0.851	0.496	0.515	0.47	0.471	0.524	0.609	6
BOK	0.432	0.34	0.294	0.211	0.043	0.539	0.699	0.431	0.369	0.373	0.458	0.37	0.38	14
BOP	1.073	1.183	0.144	0.187	0.007	0.009	0.281	0.319	0.345	0.452	0.419	0.526	0.411	12
**Mean public**	**0.686**	**0.69**	**0.463**	**0.234**	**0.145**	**0.405**	**0.528**	**0.363**	**0.34**	**0.413**	**0.421**	**0.393**	**0.423**	
ABL	0.456	0.436	0.45	0.388	0.425	0.576	1.096	0.501	0.57	0.41	0.512	0.525	0.529	9
ASBL	0.433	0.411	0.385	0.211	0.17	0.315	0.475	0.324	0.388	0.325	0.36	0.402	0.35	19
BIP	1.106	0.28	0.659	0.124	0.079	0.15	0.3	0.235	0.221	0.104	0.106	0.167	0.294	20
BAF	0.275	0.317	0.359	0.223	0.213	0.386	0.611	0.406	0.402	0.289	0.348	0.462	0.358	17
BAH	0.410	0.365	0.461	0.232	0.262	0.394	0.511	0.388	0.333	0.223	0.274	0.488	0.362	16
DIBP	0.411	0.344	1.209	0.134	0.124	0.266	0.491	0.344	0.389	0.239	0.33	0.468	0.396	13
FBL	1.006	0.43	1.377	0.303	0.277	0.324	0.472	0.379	0.377	0.321	0.442	0.451	0.513	10
HBL	0.556	0.422	0.522	0.347	0.48	0.664	0.791	0.517	0.546	0.508	0.593	0.646	0.549	8
HMBL	0.845	0.494	0.526	0.471	0.488	0.586	0.764	0.522	0.49	0.493	0.485	0.513	0.556	7
JSBL	0.102	0.16	0.292	0.107	0.113	0.363	1.018	0.4	0.488	0.393	0.478	0.358	0.356	18
MCB	1.327	1.321	1.252	1.378	1.194	1.159	1.158	1.003	0.639	1.068	1.062	0.752	1.109	2
MBL	0.389	0.404	0.295	0.293	0.33	0.423	0.595	0.437	0.458	0.321	0.525	0.567	0.42	11
SBL	0.0081	0.118	0.098	0.04	0.152	0.15	0.248	0.165	0.152	0.254	0.343	0.299	0.169	24
SKBL	0.132	0.08	0.076	0.009	0.142	0.19	0.278	0.245	0.351	0.227	0.345	0.379	0.205	23
SRB	0.404	0.340	0.448	0.28	0.233	0.426	0.521	0.391	0.379	0.32	0.294	0.369	0.367	15
SCB	0.691	0.650	1.02	0.363	0.617	0.601	0.957	1.002	1.043	1.098	1.065	1.115	0.852	4
UBL	0.552	0.507	0.568	0.413	0.47	0.71	0.979	0.607	0.677	0.513	0.704	1.007	0.642	5
ABIBP	0.282	0.29	0.247	0.122	0.096	0.235	0.286	0.255	0.27	0.179	0.263	0.213	0.228	22
**Mean private**	**0.521**	**0.409**	**0.569**	**0.302**	**0.326**	**0.44**	**0.642**	**0.451**	**0.454**	**0.405**	**0.474**	**0.51**	**0.459**	
CB NA	0.439	0.433	0.633	0.344	0.346	0.563	1.137	1.423	1.283	1.285	1.427	1.141	0.871	3
DB AG	1.388	1.51	1.466	2.132	1.678	1.473	0.818	0.676	1.333	1.397	1.508	1.552	1.411	1
**Mean foreign**	**0.914**	**0.972**	**1.05**	**1.238**	**1.012**	**1.018**	**0.978**	**1.05**	**1.308**	**1.341**	**1.468**	**1.347**	**1.141**	
**Total Mean**	**0.582**	**0.503**	**0.591**	**0.369**	**0.353**	**0.482**	**0.651**	**0.486**	**0.506**	**0.484**	**0.548**	**0.560**	**0.510**	

**Fig 4:**
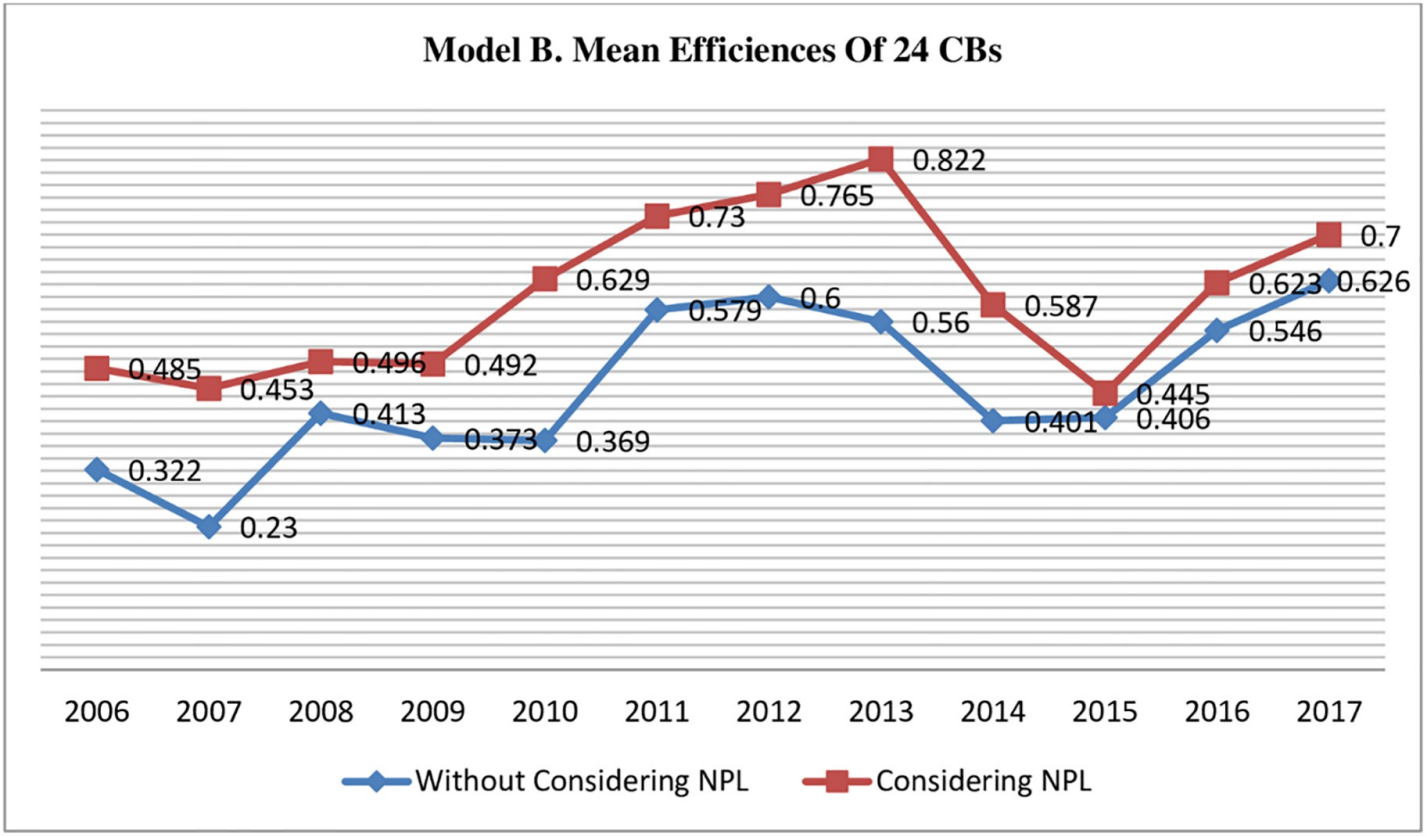
Model B comparison of mean efficiency scores of all CBs estimated with and without incorporating undesirable output NPLs.

Table 5 present the results measured through Model B, which indicate the mean efficiency score (considering NPLs) for 12 years (2006–17) is 0.602 while (without considering NPLs) is 0.452, which suggests that CBs operating in Pakistan still operate inefficiently and needs to improve their operational efficiency level. Further results indicate a considerable difference (15%) among mean efficiency scores calculated considering and without considering (bad output) NPLs (see Fig 4). Similar to model A, model B reveals that whether we ignore or consider NPLs in the efficiency estimation process, CBs’s sector-wise position doesn’t change much. As considering bad output (Foreign CBs >Private CBs >Public CBs) = (1.181 >0.581 > 0.405) while ignoring bad output (Foreign CBs >Private CBs >Public CBs) = (1.144 >0.411 > 0.29), clearly indicating that mean efficiency scores were changed while including the NPLs in outputs of Model B. Ranking the most efficient CB of each sector, it was noticed that considering NPLs as bad output, DB AG (foreign CB) was the most efficient among all 24 CBs. BAH and NBP were top for the private and public sectors. When NPLs were ignored, BAH changed its position to 22, and FBL was ranked 1^st^ among Private CBs; however, DB AG and NBP retained their positions.

**Table 5:** Model B. Efficiency of 24 CBs considering the undesirable output.

DMU	2006	2007	2008	2009	2010	2011	2012	2013	2014	2015	2016	2017	Mean	Rank
FWB	0.241	0.173	0.242	0.098	0.258	1.023	0.319	0.445	0.154	0.247	1.089	0.276	0.38	21
NBP	0.257	0.21	0.38	0.363	0.566	0.758	1.004	0.767	0.381	0.3	0.69	0.506	0.515	13
BOK	0.164	0.217	0.195	0.112	0.053	0.464	0.538	0.458	0.19	0.165	0.352	0.518	0.286	24
BOP	0.521	0.618	0.548	0.265	0.15	0.364	0.489	0.412	0.158	0.305	1.074	0.354	0.438	17
**Mean public**	**0.296**	**0.305**	**0.341**	**0.21**	**0.257**	**0.652**	**0.588**	**0.521**	**0.221**	**0.254**	**0.801**	**0.414**	**0.405**	
ABL	0.155	0.133	0.309	0.662	0.561	1.03	1.116	0.82	0.326	0.203	0.352	0.372	0.503	15
ASBL	0.331	0.23	0.333	0.175	0.307	0.417	0.51	0.439	0.253	0.205	0.518	0.404	0.344	22
BIP	0.168	0.259	0.256	0.173	0.393	0.512	0.508	1.037	1.053	0.057	0.241	0.351	0.417	20
BAF	0.417	0.393	0.262	0.185	0.499	0.391	0.791	0.978	0.297	0.228	0.516	0.741	0.475	16
BAH	0.622	1.275	1.24	1.191	1.352	1.19	1.222	1.175	1.037	0.129	0.173	1.006	0.968	2
DIBP	0.332	1.118	0.522	1.051	0.487	0.748	0.588	0.705	1.09	1.096	0.369	1.066	0.764	6
FBL	0.583	0.391	1.037	1.006	0.811	1.06	1.07	1.09	1.009	1.021	1.099	0.648	0.902	4
HBL	0.216	0.152	0.426	0.301	0.59	0.607	0.729	0.59	0.307	0.314	0.482	0.507	0.435	18
HMBL	1.564	1.122	1.115	1.089	1.135	1.022	0.765	0.815	0.306	0.273	0.467	0.38	0.838	5
JSBL	1.081	0.445	0.365	0.169	0.326	0.449	1.016	1.005	0.507	0.322	0.284	0.841	0.568	10
MCB	0.305	0.228	0.401	0.315	0.484	0.557	0.686	0.627	0.302	0.264	0.419	0.509	0.425	19
MBL	0.536	0.525	0.146	0.321	1	0.474	0.626	0.831	0.477	0.161	0.241	1.037	0.531	11
SBL	0.099	0.065	0.156	1.017	1.013	1.059	0.401	1.015	1.012	0.394	0.669	1.088	0.666	8
SKBL	0.181	0.161	0.209	0.156	1.004	0.764	0.549	1.06	1.077	1.006	1.081	1.065	0.693	7
SRB	0.509	0.348	0.431	0.289	0.461	0.954	1.002	1.035	0.479	0.294	0.506	0.84	0.596	9
SCB	0.271	0.226	0.462	0.287	1.069	0.775	0.82	0.607	0.339	0.254	0.54	0.492	0.512	14
UBL	0.246	0.19	0.457	0.68	0.572	0.698	0.759	0.737	0.357	0.273	0.604	0.619	0.516	12
ABIBP	0.175	0.268	0.372	0.199	0.209	0.312	0.341	0.394	0.244	0.255	0.392	0.584	0.312	23
**Mean private**	**0.433**	**0.418**	**0.472**	**0.515**	**0.682**	**0.723**	**0.75**	**0.831**	**0.582**	**0.375**	**0.497**	**0.697**	**0.581**	
CB NA	1.173	0.585	0.525	0.282	0.438	0.536	1.062	1.25	1.126	1.074	1.729	1.091	0.906	3
DB AG	1.494	1.547	1.524	1.432	1.349	1.367	1.441	1.429	1.612	1.839	1.074	1.505	1.468	1
**Mean foreign**	**1.334**	**1.066**	**1.025**	**0.857**	**0.894**	**0.952**	**1.252**	**1.34**	**1.369**	**1.457**	**1.402**	**1.298**	**1.187**	
**Total Mean**	**0.485**	**0.453**	**0.496**	**0.492**	**0.629**	**0.73**	**0.765**	**0.822**	**0.587**	**0.445**	**0.623**	**0.7**	**0.602**	
Model B. Efficiency of 24 CBs ignoring undesirable output														
FWB	0.069	0.06	0.144	0.035	0.053	0.464	0.18	0.22	0.115	0.225	0.415	0.246	0.186	23
NBP	0.229	0.169	0.35	0.291	0.217	0.704	0.909	0.761	0.35	0.284	0.458	0.516	0.437	8
BOK	0.153	0.205	0.162	0.083	0.012	0.403	0.51	0.265	0.153	0.149	0.288	0.439	0.235	20
BOP	0.226	0.225	0.547	0.21	0.096	0.321	0.493	0.382	0.122	0.27	0.4	0.319	0.301	17
**Mean public**	**0.169**	**0.165**	**0.301**	**0.155**	**0.095**	**0.473**	**0.523**	**0.407**	**0.185**	**0.232**	**0.39**	**0.38**	**0.29**	
ABL	0.113	0.097	0.248	0.463	0.251	0.579	0.731	0.416	0.274	0.166	0.344	0.303	0.332	14
ASBL	0.154	0.18	0.272	0.127	0.088	0.331	0.478	0.365	0.216	0.171	0.415	0.371	0.264	18
BIP	0.147	0.079	0.146	0.058	0.039	0.114	0.2	0.209	0.093	0.041	0.111	0.316	0.129	24
BAF	0.137	0.145	0.199	0.093	0.13	0.38	0.553	0.458	0.235	0.182	0.565	0.679	0.313	15
BAH	0.158	0.133	0.255	0.061	0.077	0.222	0.348	0.28	0.128	0.098	0.224	0.351	0.195	22
DIBP	0.059	0.117	0.211	0.5	0.294	0.418	0.417	0.338	0.369	1.066	0.525	1.034	0.446	7
FBL	0.368	0.248	1.05	1.009	0.962	1.094	1.109	1.142	1.014	1.032	1.042	0.692	0.897	2
HBL	0.174	0.113	0.366	0.231	0.236	0.525	0.569	0.402	0.266	0.291	0.42	0.481	0.34	13
HMBL	1.132	0.22	1.068	1.071	1.089	0.913	0.772	0.629	0.271	0.265	0.389	0.333	0.679	5
JSBL	1.127	0.181	0.304	0.074	0.081	0.382	0.643	0.546	0.369	0.286	0.473	0.642	0.426	12
MCB	0.157	0.125	0.324	0.225	0.138	0.403	0.532	0.385	0.253	0.238	0.457	0.513	0.313	15
MBL	0.184	0.15	0.111	0.088	0.138	0.265	0.347	0.31	0.172	0.125	0.383	0.567	0.237	19
SBL	0.096	0.052	0.126	1.025	1.012	1.07	0.324	1.006	0.386	0.358	0.659	1.138	0.604	6
SKBL	0.153	0.129	0.168	0.118	1.006	0.785	0.558	1.082	1.12	1.009	1.108	1.075	0.693	4
SRB	0.16	0.132	0.378	0.182	0.218	0.749	0.575	0.803	0.38	0.253	0.437	0.882	0.429	11
SCB	0.193	0.186	0.478	0.227	0.594	0.776	0.825	0.517	0.323	0.244	0.357	0.505	0.435	10
UBL	0.176	0.142	0.409	0.594	0.22	0.624	0.743	0.578	0.323	0.247	0.579	0.606	0.437	8
ABIBP	0.138	0.13	0.267	0.148	0.042	0.239	0.267	0.266	0.182	0.21	0.286	0.642	0.235	20
**Mean private**	**0.268**	**0.142**	**0.354**	**0.35**	**0.368**	**0.548**	**0.555**	**0.541**	**0.354**	**0.349**	**0.487**	**0.618**	**0.411**	
CB NA	0.28	0.232	0.483	0.218	0.221	0.58	1.094	1.333	1.201	1.109	1.647	1.113	0.793	3
DB AG	1.94	2.069	1.854	1.827	1.633	1.549	1.213	0.758	1.316	1.415	1.115	1.249	1.495	1
**Mean foreign**	**1.11**	**1.151**	**1.169**	**1.023**	**0.927**	**1.065**	**1.154**	**1.046**	**1.259**	**1.262**	**1.381**	**1.181**	**1.144**	
**Total Mean**	**0.322**	**0.23**	**0.413**	**0.373**	**0.369**	**0.579**	**0.6**	**0.56**	**0.401**	**0.406**	**0.546**	**0.626**	**0.452**	

Efficiency results estimated through two different input-output bundles demonstrated that changing the inputs-outputs bundles doesn’t change the position (rank) of different commercial banking sectors and oppose the research results by [42] in Pakistan banking setup. However, there is considerable variation in annual mean efficiency scores calculated from 2 different inputs-outputs bundles. In addition, the top-ranked CB of each sector doesn’t change its position except private CBs, which have a higher level of NPLs than foreign or public CBs (see Fig 2), showing the impact of bad output on efficiency level.

### 4.2 Efficiency comparison of Big 5 CBs with other domestic CBs

The top market share of the banking sector in Pakistan is controlled by five big banks (HBL, NBP, UBL, MCB, and ABL). These five banks account for 56% of the country’s deposits and 52% of the country’s banking industry’s advances. Table 6 shows the efficiency comparison of Big 5 CBs with 1**7** other domestic CBs. Results of super-efficiency estimated through model A shows that Mean efficiency scores (irrespective of undesirable output) of big 5 are higher than all other 17 CBs, (0.686 >0.492) (0.687 >0.382). However, Model B presents the converse results, where the mean efficiency of big 5 CBs is less than the other 17 domestic CBs (0.478<0.57) (0.372<0.394).

**Table 6:** Efficiency comparison between Big 5 CBs and other domestic CBs.

**Model A. considering the undesirable output**														
**DMU**	**2006**	**2007**	**2008**	**2009**	**2010**	**2011**	**2012**	**2013**	**2014**	**2015**	**2016**	**2017**	**Mean**	**rank**
HBL	0.457	0.403	0.5	0.37	0.616	0.608	0.716	0.463	0.551	0.567	0.652	0.747	0.554	11
NBP	0.774	0.613	0.648	0.392	0.53	0.56	0.769	0.456	0.43	0.436	0.492	0.49	0.549	12
UBL	0.511	0.446	0.528	0.42	0.629	0.625	0.873	0.553	0.638	0.601	0.748	1.007	0.632	9
MCB	1.204	1.222	1.155	1.233	1.154	1.118	1.124	1.03	1.059	1.08	1.085	0.784	1.104	3
ABL	0.38	0.377	0.414	0.405	0.635	0.552	1.076	0.485	0.602	0.547	0.635	1.013	0.593	10
**Mean Big 5**	**0.665**	**0.612**	**0.649**	**0.564**	**0.712**	**0.6926**	**0.911**	**0.597**	**0.656**	**0.646**	**0.722**	**0.808**	**0.686**	
**Mean Others**	**0.679**	**0.637**	**0.585**	**0.279**	**0.453**	**0.362**	**0.550**	**0.431**	**0.461**	**0.422**	**0.498**	**0.545**	**0.492**	
**Model A. without considering the undesirable output**														
HBL	0.556	0.422	0.522	0.347	0.48	0.664	0.791	0.517	0.546	0.508	0.593	0.646	0.549	8
NBP	0.923	0.727	0.767	0.437	0.456	0.673	0.851	0.496	0.515	0.47	0.471	0.524	0.609	6
UBL	0.552	0.507	0.568	0.413	0.47	0.71	0.979	0.607	0.677	0.513	0.704	1.007	0.642	5
MCB	1.327	1.321	1.252	1.378	1.194	1.159	1.158	1.003	0.639	1.068	1.062	0.752	1.109	2
ABL	0.456	0.436	0.45	0.388	0.425	0.576	1.096	0.501	0.57	0.41	0.512	0.525	0.529	9
**Mean Big 5**	**0.762**	**0.682**	**0.711**	**0.592**	**0.605**	**0.756**	**0.975**	**0.624**	**0.589**	**0.593**	**0.668**	**0.690**	**0.687**	
**Mean Others**	**0.489**	**0.395**	**0.502**	**0.200**	**0.201**	**0.338**	**0.516**	**0.379**	**0.387**	**0.351**	**0.404**	**0.429**	**0.382**	
**Model B. considering the undesirable output**														
HBL	0.216	0.152	0.426	0.301	0.59	0.607	0.729	0.59	0.307	0.314	0.482	0.507	0.435	18
NBP	0.257	0.21	0.38	0.363	0.566	0.758	1.004	0.767	0.381	0.3	0.69	0.506	0.515	13
UBL	0.246	0.19	0.457	0.68	0.572	0.698	0.759	0.737	0.357	0.273	0.604	0.619	0.516	12
MCB	0.305	0.228	0.401	0.315	0.484	0.557	0.686	0.627	0.302	0.264	0.419	0.509	0.425	19
ABL	0.155	0.133	0.309	0.662	0.561	1.03	1.116	0.82	0.326	0.203	0.352	0.372	0.503	15
**Mean Big 5**	**0.235**	**0.182**	**0.394**	**0.464**	**0.554**	**0.73**	**0.858**	**0.708**	**0.334**	**0.27**	**0.509**	**0.502**	**0.478**	
**Mean Others**	**0.458**	**0.46**	**0.464**	**0.457**	**0.619**	**0.704**	**0.679**	**0.794**	**0.569**	**0.377**	**0.565**	**0.687**	**0.57**	
**Model B. without considering the undesirable output**														
HBL	0.174	0.113	0.366	0.231	0.236	0.525	0.569	0.402	0.266	0.291	0.42	0.481	0.34	13
NBP	0.229	0.169	0.35	0.291	0.217	0.704	0.909	0.761	0.35	0.284	0.458	0.516	0.437	8
UBL	0.176	0.142	0.409	0.594	0.22	0.624	0.743	0.578	0.323	0.247	0.579	0.606	0.437	8
MCB	0.157	0.125	0.324	0.225	0.138	0.403	0.532	0.385	0.253	0.238	0.457	0.513	0.313	15
ABL	0.113	0.097	0.248	0.463	0.251	0.579	0.731	0.416	0.274	0.166	0.344	0.303	0.332	14
**Mean Big 5**	**0.169**	**0.129**	**0.339**	**0.361**	**0.212**	**0.567**	**0.697**	**0.508**	**0.293**	**0.245**	**0.452**	**0.484**	**0.372**	
**Mean Others**	**0.273**	**0.151**	**0.346**	**0.301**	**0.349**	**0.525**	**0.506**	**0.519**	**0.332**	**0.352**	**0.475**	**0.602**	**0.394**	

### 4.3 Impact of NIM, Bank size and NPLs on the efficiency of Pakistan’s CBs

After efficiency analysis, we have applied the system GMM with two steps to make our study more comprehensive and conclusive, regrinding the concerned variables’ impacts. The standard diagnostic tests (AR (1), AR(2), Hansen tests) are also included to ensure that the applied method is suitable for the study. Further, the joint validity of the lagged instrumental variables is certified by the Hansen values. Second, the diagnostic tests reveal no significant evidence of serial correlation in the first-differenced errors at order 2. Typically, undesirable output has been ignored while estimating the banking efficiency. But, the present research incorporated the undesirable output in determining the banking efficiency and regressed the regression to confirm the robustness of the findings. The results extracted from Eq (3) are reported in Table 7. Banking efficiency has been measured in two ways using different measures as mentioned in section (2). Model (1) showed that bank size positively contributes to increasing Pakistan’s banking efficiency as the coefficient (0.327) is statistically positive and significant at a 1 percent level. The positivity of bank size to increase banking efficiency is consistent with [114, 115], who stated that bank size is essential for determining banking efficiency.

**Table 7:** System—GMM results.

	Model (1)	Model (2)
Variables	BE	BE
L.BE	-0.442***	
	(0.0679)	
L.BE		0.571***
		(0.0580)
Bank _ size	0.327***	0.215***
	(0.0980)	(0.0570)
NIM	0.0796***	0.0997**
	(0.0295)	(0.0463)
NPL	-0.281***	-0.170***
	(0.0901)	(0.0366)
Constant	-1.257*	-1.480**
	(0.707)	(0.742)
AR (1)	-1.92	-3.06
	[0.055]	[0.002]
AR(2)	-1.18	-1.86
	[0.238]	[0.063]
Hansen test [prob.]	[0.112]	[0.252]
Observations	264	264
Numbers of firms	24	24

Note: Standard errors in parentheses*** p<0.01, ** p<0.05, * p<0.1. Prob. value are given in brackets.

Likewise, we found that the net interest margin significantly increases banking efficiency. The results implied that the net interest margin is the potential measure to uplift the level of efficiency of the Pakistani banking industry. [116] suggested that net interest margin is the utmost appropriate criterion for assessing the stability of banks’ operations.Net interest margin, which is the difference between banks’ lending and deposit rates, is the essential and primary indicator in the financial system [117, 118].

On the other hand, non-performing loans and banking efficiency are negatively linked in columns (1). It means that non-performing loans degrade the efficiency level of the Pakistani banking industry. Our results are validated by [119, 120], who stated that the non-performing loans probably induce the inefficiency of the banking industry. Further, [98, 121] made arguments that grating non-performing loans is the result of bad management that affects the banking efficiency level. Because of poor evaluation services, bankers do not perform their duties efficiently. They might not correctly evaluate the customers’ credit applications, leading to lower credit ratings for the approved loans and a high chance of defaulting in arrears to higher non-performing loans. We regressed the concerned explanatory variables on the other two banking efficiency variables for robustness. The results of model (2) are reported in Table 6, which validated that bank size and net interest margin are positive parameters to improve Pakistan’s banking efficiency. However, non-performing loans are found to be detrimental to banking efficiency in Model (2).

## 5. Conclusion and policy implication

This research aimed to treat NPLs as an undesirable output in the efficiency evaluation of commercial banks, gauge the change in result output, and further differentiate the efficiency level among three different banking sectors (public, private and foreign) of Pakistan. In addition, efficiency comparison of big 5 CBs with other domestic CBs included in the research to distinguish the efficiency level of big CBs from others reaming. To this end, we applied the DEA Super-SBM model incorporating NPLs as bad output, with two different input-output bundles, namely model A and B. 24 CBs of Pakistan were evaluated for 12 years, ranging from 2006-to 2017. To prove the creditability of our results, in the second stage of our analysis, we applied system GMM to regress banking efficiency on NPLs, bank size, and NIM. Results indicate that for both models, there is considerable change noticed in super efficiency scores estimated through incorporating NPLs in input-output bundles of CBs, compared to the efficiency scores measured without incorporating NPLs in input-output bundles. Model A and B indicate that foreign banks are always more efficient than Local Banks, while private banks have greater efficiency scores than public banks in domestic banking.

Moreover, five big CBs reveal mixed results, as in model A estimation, they were more efficient than other domestic CBs. In contrast, they were less efficient in model B than their counterparts. System GMM results proved that NPL is negatively and significantly associated with CBs efficiency in the second stage of empirical analysis. Bank size and net interest margin are positively associated with CBs efficiency. Because of poor evaluation services, bankers do not perform their duties efficiently. They might not properly evaluate the customers’ credit applications, leading to lower credit ratings for the approved loans and a high chance of defaulting in debts to higher non-performing loans. Therefore, it is advised to the managers and policymakers of CBs that after minimizing the NPLs ratio, they could make their banks more efficient and stable. Domestic CBs (public, private) are suggested to follow the operational strategies of foreign banks as they are most efficient among all. Although NPLs ratio of private banks is higher than public CBs, they are more efficient than public CBs, suggesting that public CBs need to improve their income level and reduce cost through improved operational strategies and better service quality. As bank size also improves Pakistan’s CBs’s banking efficiency, small CBs can expand their deposit scale to higher operational efficiency. Data availability and time constraint are limitations of our study; after efficiency estimation, more contextual variables could be regressed to explore the effect of those variables on banking efficiency.

## Supporting information

S1 Table List of CBs. (DOCX)
