# Supplementary material for: Efficiency evaluation of commercial banks in Pakistan: A slacks-based measure Super-SBM approach with bad output (Non-performing loans)
Source: PLoS One. 2022 Jul 12;17(7):e0270406. doi: 10.1371/journal.pone.0270406 (PMC9275710; doi:10.1371/journal.pone.0270406)
Supplement: S1 Table — (DOCX) [file pone.0270406.s001.docx]

**Table S1. List of CBs**

| No | CB name | Abbreviations |
| --- | --- | --- |
| 1 | First Women Bank Limited | FWB |
| 2 | National Bank of Pakistan | NBP |
| 3 | The Bank Of Khyber | BOK |
| 4 | The Bank Of Punjab | BOP |
| 5 | Allied Bank Ltd. | ABL |
| 6 | Askari Bank Ltd. | ASBL |
| 7 | Bankislami Pakistan Ltd. | BIP |
| 8 | Bank Al-Falah Ltd. | BAG |
| 9 | Bank Al-Habib Ltd. | BAH |
| 10 | Dubai Islamic Bank Pakistan Ltd. | DIP |
| 11 | Faysal Bank Ltd. | FBL |
| 12 | Habib Bank Ltd | HBL |
| 13 | Habib Metropolitan Bank Ltd. | HMBL |
| 14 | JS Bank Ltd. | JSBL |
| 15 | MCB Bank Ltd. | MCB |
| 16 | Meezan Bank Ltd. | MBL |
| 17 | SAMBA Bank Ltd. | SBL |
| 18 | Silk Bank Ltd. | SKBL |
| 19 | Soneri Bank | SRB |
| 20 | Standard Chartered Bank | SCB |
| 21 | United Bank Ltd. | UBL |
| 22 | Alberta Islamic Bank (Pakistan) | ABIB |
| 23 | Citi Bank NA | CB NA |
| 24 | Deutch Bank AG | DB AG |
